# α-Mangostin/γ-Cyclodextrin Inclusion Complex: Formation and Thermodynamic Study

**DOI:** 10.3390/polym13172890

**Published:** 2021-08-27

**Authors:** Ine Suharyani, Muchtaridi Muchtaridi, Ahmed Fouad Abdelwahab Mohammed, Khaled M. Elamin, Nasrul Wathoni, Marline Abdassah

**Affiliations:** 1 Department of Pharmaceutics and Pharmaceutical Technology, Faculty of Pharmacy, Universitas Padjadjaran, Sumedang 45363, Indonesia; ine18001@mail.unpad.ac.id; 2 School of Pharmacy Muhammadiyah Cirebon, Cirebon 45153, Indonesia; 3 Department of Pharmaceutical Analysis and Medicinal Chemistry, Faculty of Pharmacy, Universitas Padjadjaran, Sumedang 45363, Indonesia; muchtaridi@unpad.ac.id; 4 Department of Pharmaceutics, Faculty of Pharmacy, Minia University, Minia 61519, Egypt; Ahmed.mohamed1@minia.edu.eg; 5 Global Center for Natural Resources Sciences, Faculty of Life Sciences, Kumamoto University, Kumamoto 862-0973, Japan; khaled@kumamoto-u.ac.jp

**Keywords:** α-Mangostin, γ-cyclodextrin, inclusion complex, solubility, thermodynamic study

## Abstract

α-Mangostin (α-M) has various biological activities, such as anti-cancer, antibacterial, anti-fungal, anti-tyrosin, anti-tuberculosis, anti-inflammatory, and antioxidant. However, it has very low solubility in water. The formulation of this compound requires high amounts of solubilizers, which limits its clinical application. In addition, its low solubility in water is a barrier to the distribution of this drug, thus affecting its potency. Cyclodextrin (CD) is widely used as a solubility enhancer of poorly soluble drugs. This study aimed to increase the solubility of α-M in water through complex formation with CD. The complex of α-Mangostin and γ-Cyclodextrin (α-M/γ-CD CX) was prepared by the solubilization method, resulting in a solubility improvement of α-M in water. Characterization of α-M/γ-CD CX by using FTIR-Spectrometry, XRD, H-, C-, and HMBC-NMR showed that α-M was able to form an inclusion complex with γ-CD. The complex yielded an entrapment efficiency of 84.25 and the thermodynamic study showed that the α-M/γ-CD CX was formed spontaneously, based on the negative values of Gibbs energy and ΔH. Interestingly, the solubility of α-M/γ-CD CX significantly increased by 31.74-fold compared with α-M. These results suggest that α-M/γ-CD CX has the potential in the formulation of water-based preparation for clinical applications.

## 1. Introduction

**Figure 1:**
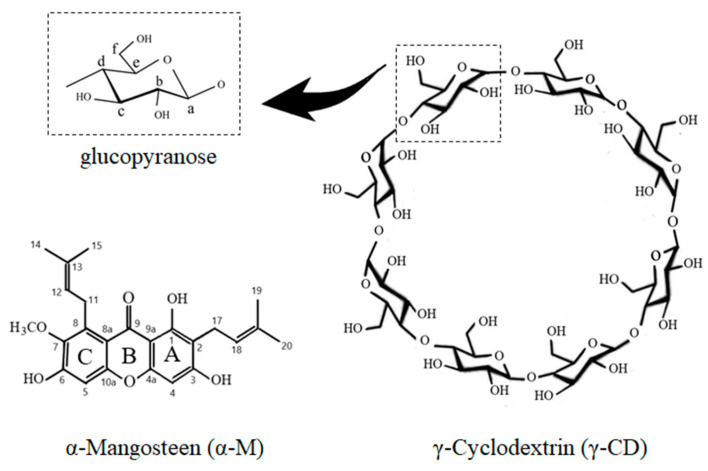
Chemical structures of α-Mangostin (α-M), γ-Cyclodextrin (γ-CD), and glucopyranose (the monomer of γ-CD).

α-Mangostin [1, 3, 6-trihydroxy-7-methoxy-2, 8-bis (3-methylbut-2-enyl) -9H-xanthen-9-one] (α-M) is a major compound in *Garcinia mangostana* pericarp extract (Figure 1) [1]. α-M is widely used for anti-cancer, antibacterial, anti-fungal, anti-tyrosin, anti-tuberculosis, anti-inflammatory, and antioxidant [2,3,4,5,6]. Despite the various pharmacological effects of α-M, its poor solubility in water limits its application. Several studies have been undertaken to increase α-M solubility in water, with the solubility enhancement of α-M being performed by complex formation, co-solvent, or nanomicelle formation [1,7,8,9]. α-M nanomicelles were found to increase the solubility from 0.2 ± 2 μg/mL to about 2743 ± 11 μg/mL [7]; α-M/β-CD was made with the addition of ethanol as a co-solvent, which increased the affinity of α-M to the β-CD molecule [9]; another study simulated the permeation enhancement of the α−M/β-CD and α−M/2,6-dimethyl-β-CD complex [10]. In addition, the α−M/hydroxypropyl-β-CD complex successfully improved water solubility and wound healing activity [1,3]. An in silico study of the inclusion complex α-M with α-, β-, and γ-CD by using a computational simulation showed that the most favorable complex was α-M/γ-CD, which has the smallest ∆G [1]; however, experimental data which use γ-CD as a host for α-M have not yet been reported.

Cyclodextrin (CD) is an oligosaccharide molecule that has a unique cavity structure: it has a hydrophobic property in the inner cavity but is hydrophilic in the outer cavity. The conical truncated structure of CDs proposed to include the hydrophobic molecule in the cavity, resulting in their improved water solubility. CD molecules are formed by glucopyranose units via α-1,4-glycosidic linkages. The type of CDs correlated with the number of glucopyranose units. α-, β-, and γ-CD contain six, seven, and eight units, respectively [11]. Some new types of CDs were synthetized from three and four glucopyranose units [12]. γ-CD is a cyclodextrin consisting of eight glucopyranose units (Figure 1) [13] and providing a larger cavity than other types of CDs. A number of studies using CDs inclusion complexes have been reported for water solubility enhancement. The inclusion complex of α-, β- CD with 5-fluorouracil buprofen [14,15], HP-β-CD with thiophanante-methyl, thiram, difenoconazole [16,17,18], and γ-CD with forchlorfenuron were formed and increased their water solubility [19,20].

In this study, the inclusion complexes of α-M and γ-CD were prepared by solubilization method to improve the solubility of α-M in water. The phase solubility study was implemented to display the solubility enhancement of α-M in several concentrations of γ-CD and a Job plot predict the molar ratio of the complex formation. Several characterizations such as FTIR-Spectrometry, XRD, SEM, H-, C-, and HMBC-NMR were performed to study the configuration of the inclusion complex. The termodynamic studies were implemented to study the complex formation process. α-M could be more widely used both in formulation and several pharmacological applications due to the increased solubility of α-M in the inclusion complex.

## 2. Materials and Methods

### 2.1. Materials

α-Mangostin was purchased from Chengdu Pharmaceutical Industries, China. γ-CD was a kind gift from Kumamoto University, Kumamoto, Japan. Ethanol 95% and aqua deionized to a high purity were purchased from Sigma Aldrich, St. Louis, MO, USA. All the reagents were of analytical grade and were used without any further purification.

### 2.2. Methods

#### 2.2.1. Preparation of α-Mangostin and γ-Cyclodextrin Physical Mixture (α-M/γ-CD PM)

α-M and γ-cyclodextrin were weighed in a 1:2 molar ratio and mixed for 1 min using a vortex mixer [19,21].

#### 2.2.2. Stoichiometry Determination of α-M/γ-CD CX Formation

The stoichiometry of the α-M/γ-CD complex (α-M/γ-CD CX) formation was determined using the continuous variation method (Job plot). Each concentration (0–1 mM) of the α-M and γ-CD solution was mixed in a constant molar fraction. All the mixtures were stored at room temperature for 24 h. After equilibration, the solution was filtered using a 0.45 μm filter membrane [21,22]. The α-M in each solution was measured using an Analytic Jena Specord 200 at 316 nm [22].

#### 2.2.3. Phase Solubility Studies

This study was performed according to The Higuchi and Connors Method [23]. An excess of α-M (25 mg) was added into 5 mL of γ-CD solution (0, 2, 4, 6, 8, and 10 mM). Each mixture was shaken continuously in an incubator shaker at 24 °C for 96 h until equilibrium was reached [19,21,24,25]. The mixture was filtered using a 0.45 µm filter membrane, and the soluble α-M was measured using UV-vis spectrometry at 316 nm [22].

The solubility improvement of α-M/γ-CD CX in water was investigated by dispersing 0.1–0.3 g of α-M, α-M/γ-CD PM, and α-M/γ-CD CX in 10 mL of distilled water. The mixtures were shaken for 24 h at 25 °C. The mixtures were then filtered with 0.45 μm filter membrane. The concentration of α-M was measured using an Analytic Jena Specord 200 at 316 nm [22].

#### 2.2.4. Preparation of α-Mangostin and γ-Cyclodextrin Complex (α-M/γ-CD CX)

The complex was prepared by the solubility method. A total amount of 1 mM of α-M solution in ethanol 95% and 2 mM of γ-CD solution in water was made in a separated volumetric flask. α-M solution was gradually added into γ-CD solution and mixed for about 24 h at room temperature. Then, the solution was evaporated at 60 °C to yield the complex powder. The entrapment efficiency of the complex was measured by an Analytic Jena Specord 200 at 316 nm [8,9,22,26].

#### 2.2.5. Characterization of α-Mangostin (α-M), γ-Cyclodextrin (γ-CD), Physical Mixture (α-M/γ-CD PM), and Inclusion Complex (α-M/γ-CD CX)

##### FTIR Spectrometry

The samples of the complex were analyzed using IR Prestige-21 Spectrometry at 400–4000 cm^−1^. About 2 mg of sample was blended with 200 mg KBr powder. The mixture was pressed into a disc and analyzed at 400–4000 cm^−1^ [19,26].

##### X-ray Diffractometry (XRD)

The XRD pattern of α-M, γ-CD, the physical mixture (α-M/γ-CD PM), and the inclusion complex (α-M/γ-CD CX) were analyzed using a XRD Bruker D8 Advance 3 kW with LynxEye XE-T detector and radiation source CuK alfa. Each sample was scanned at 2θ from 5–50 [7,19,26].

##### Scanning Electron Microscopy (SEM)

The morphology surface of α-M, γ-CD, α-M/γ-CD PM, and α-M/γ-CD CX were assessed by scanning electron microscopy (SEM) JEOL JSM 6510 LA. The sample was placed in a sample holder, sprayed to remove impurities, and coated with gold. The analyses were carried out at an acceleration voltage of 15 kV, and each sample was observed at 250 and 3000 magnification [19,26].

##### NMR Spectrometry

The structure of α-M, γ-CD, α-M/γ-CD PM and α-M/γ-CD CX were analyzed using NMR spectrometry 500 Hz JEOL, JNM ECA 500 [19,27].

#### 2.2.6. Thermodynamic Study of α-M/γ-CD CX Formation

An excess of α-M (±25 mg) was added into 5 mL γ-CD solution (0, 2, 4, 6, 8, and 10 mM). Each mixture was shaken continuously in an incubator shaker at 24 °C for 96 h until equilibrium was reached [19,21,24]. The mixture was filtered using a 0.45 µm filter membrane, and the soluble α-M was measured using UV-vis spectrometry at 316 nm [22]. This procedure was carried out at temperatures of 25 °C, 31 °C, and 37 °C. The stability constant (*K_s_*) was calculated by using the slope value from the solubility phase diagram, utilizing the following equation:

(1) $$K_{s=\frac{slope}{S_{0}\;\left(1-slope\right)}\;}$$

*S*_0_ is the solubility of α-M without the presence of cyclodextrin, and slope is the gradient of the solubility phase diagram [19,24].

##### Determination of Enthalpy (∆*H*) and Gibbs Energy (∆*G*)

The change of enthalpy (∆*H*), and Gibbs energy (∆*G*) of α-M/γ-CD CX formation were determined using the stability constant (*Ks*) vs. temperature, following the Van’t Hoff equation:

(2) $$ln\;ln\;K{\;}_{s}=-\frac{\Delta H}{RT}$$

*K_s_* is the stability constant, *T* is the temperature (Kelvin), *R* is the gas constant (8.314 J/mL/K), and ∆*H* (enthalpy change) is calculated using the slope value from the *K_s_* vs. 1/T graph [19,24].

Furthermore, the value of ∆*G* was calculated by the following equation:

(3) $$\Delta G=-RT\;ln\;ln\;K_{s1:1}$$

#### 2.2.7. Data Analysis

Statistical comparison was performed by using Scheffe’s test. *p*-Value ≤ 0.05 is considered as statistically significant between-group population.

## 3. Results and Discussion

### 3.1. Phase Solubility Studies

A phase solubility study was undertaken to analyze the molar ratio of α-M and γ-CD in the complex α-M/γ-CD CX was prepared by the solubilization method: α-M was dissolved in ethanol, while γ-CD was dissolved in water. Both of the solutions were mixed for 24 h and evaporated at around 60 °C. The entrapment efficiency of this complex was 84.25 ± 6.80%.

**Figure 2:**
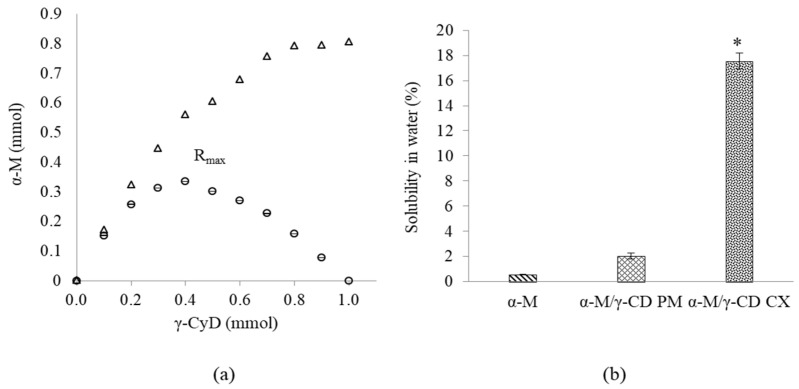
Diagrams of the Job plot and phase solubility phase (**a**) and solubility studies of αM /γ-CD (**b**). The results are expressed as the mean ± SD (*n* = 3). * *p* < 0.05, compared with αM.

The phase solubility study can be seen in Figure 2a. The phase solubility of α-M in γ-CD, following the A_L_ type of solubility diagram, indicated that α-M solubility was reached linearly with γ-CD [23,28]. At the small concentration of γ-CD (0.1 mmol) the absorbance was 0.01 ± 0.0002, and at the highest concentration of γ-CD (1 mmol) the absorbance was 0.80 ± 0.0007. This indicated that the increase in γ-CD concentration resulted in an increase in the solubility of α-M. Surprisingly, Figure 2b describes that solubility of α-M/γ-CD CX significantly improved 31.74-fold compared with α-M. In previous studies, the solubility in water increased due to molecular interaction which occurred from the inclusion complex by solubilization method [29].

The number of α-M to form the complex results in the improvement of its solubility in water. The high percentage of artemisinin complexed with γ-CD was higher than α- and β-CD [30]. Molecular docking using PM7 showed that γ-CD is the most favorable cyclodextrin to form a complex with α-M [1], and the larger diameter of the CD cavity is more suitable for the size and geometry of the guest molecule fitted to the host cavity [31]. Figure 2 indicated that α-M solubility has been reached linearly with γ-CD [24,32].

### 3.2. Stoichiometry Determination of α-M/γ-CD CX Complex Formation

The stoichiometry of complex formation was established using a Job plot. A gradient concentration of α-M was mixed with a gradient concentration of γ-CD in a constant mole fraction, and the mixture was allowed to stand overnight. The mixture was filtered by 0.45 μm membrane, and the α-M in the filtrate was measured at 316 nm. In Figure 2 (the thick circle mark), the Rmax 0.4 indicated that the stoichiometry ratio of the host (γ-CD) and guest (α-M) was 1:1 [19,24].

The toroidal structure of γ-CD can entrap α-M in its cavity [33]. The stoichiometry determination was performed by the continuous variation method/Job plot, using α-M as a guest molecule and γ-CD as a host. The result of this study is shown in the second graph (Figure 2, the thick circle mark). This study gives the R_max_ value at 0.4, corresponding with the stoichiometry ratio of the complex formation at 1:1. This ratio implies the inclusion of a single guest molecule entrapped with one molecule of the host [24,33].

### 3.3. Characterization of α-M, γ-CD, the Physical Mixture (α-M/γ-CD PM), and the Inclusion Complex (α-M/γ-CD CX)

#### 3.3.1. FTIR Spectrometry

**Figure 3:**
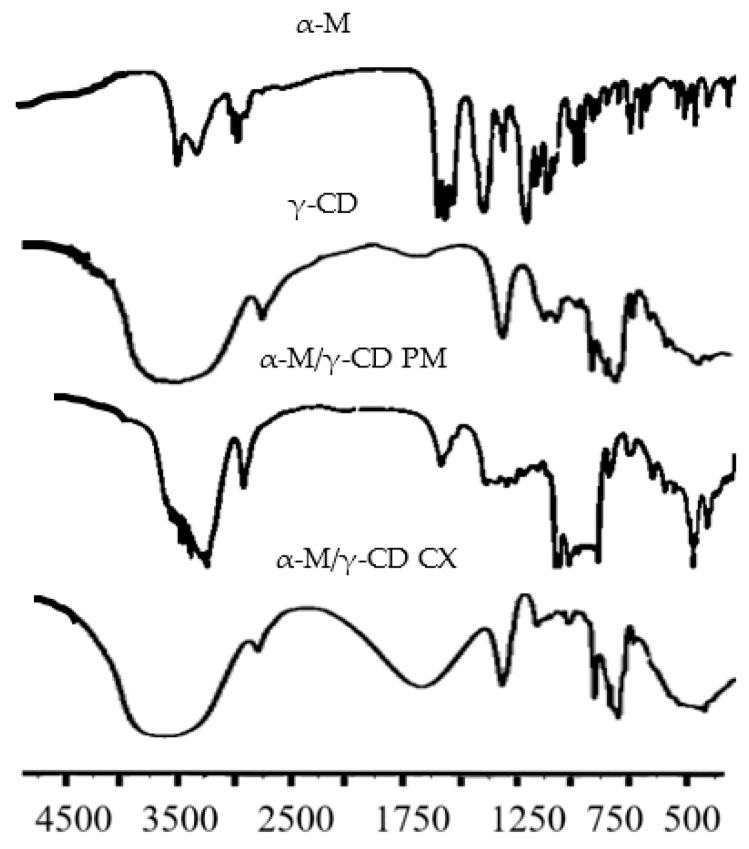
FTIR spectrum of α-M, γ-CD, α-M/γ-CD PM, and α-M/γ-CD CX.

The functional group analysis of α-M, γ-CD, α-M/γ-CD PM, and α-M/γ-CD CX was carried out using FTIR spectrophotometry at wavenumber 4500–500 cm^−1^. The FTIR spectra of α-M, γ-CD, α-M/γ-CD PM, and α-M/γ-CD CX are shown in Figure 3. The absorption band at 1600–1700 is the stretching vibration of the carbonyl group (C=O), while the strong band at 3000–3500 cm^−1^ corresponds to the hydrogen bond of the hydroxyl groups, and the weak absorption near 2900 cm^−1^ is related with sp3 hybridization of aliphatic carbon. The bend absorption at 1300–1000 cm^−1^ is the absorption of C-O groups, and the slight absorption at wavenumber 1600–1700 cm^−1^ is the vibration of C=C groups.

The FTIR spectra of α-M, γ-CD, α-M/γ-CD PM, and α-M/γ-CD CX are shown in Figure 3. Analyses of functional groups in each sample were performed at wavenumber 4500–500 cm^−1^. For interpretation, in the first graph (α-M), the stretching vibration at 3410 and 3252 cm^−1^ corresponds with the –OH in α-M. The band at 2970 and 2931 cm^−1^ is given by the asymmetric stretching vibration of sp3 hybridization of the aliphatic carbons of the α-M methyl (CH_3_) and methylene (CH_2_) groups. The absorption bands at 1580 cm^−1^, 1645 cm^−1^, and 1630 cm^−1^ are the stretching vibrations of unconjugated C=C in α-M. The band at 1470 cm^−1^ is given by the unconjugated carbonyl group (C=O) on the B ring of α-M; 1362 cm^−1^ is given by CH_3_ bending; 1083 cm^−1^ and 1051 cm^−1^ correspond with the stretching vibration of C-O; and 1011 cm^−1^, 862 cm^−1^, 804 cm^−1^, 772 cm^−1^, and 598 cm^−1^ are given by C-OH stretching, –CH=CH- (trans), –CH=CH- (cis), CH_2_, and –CH=CH- bending, respectively [34].

The second graph (γ-CD) shows a broad absorption around 3000–3600 cm^−1^ with a maximum absorption at 3395 cm^−1^. This band is related to the stretching vibration of the –OH groups in the glucose ring. The band at 2930 cm^−1^ comes from the stretching vibrations of the C-H bonds in the CH_2_. The high-intensity bands at 1030 cm^−1^ and 999 cm^−1^ are related to the C-H out-of-plane bending vibrations and the C-O stretching vibration modes, respectively [35].

The same absorption-type can be seen for the α-M/γ-CD PM (the third graph). The strong band at 3000–3500 cm^−1^ corresponds to the hydrogen bond of the hydroxyl groups. The weak absorption 2930 cm^−1^ corresponds with the methylene (CH_2_) groups, 1640 cm^−1^ is given by CH_3_ bending, 998 cm^−1^ is the bend absorption of C-OH stretching vibration, and 598 cm^−1^ indicates –CH=CH- bending [34,35].

The last graph is the spectra for α-M/γ-CD CX. The broad band around 3000–3600 cm^−1^ with the maximum absorption at 3346 cm^−1^ is given by the stretching vibration of the –OH groups in the glucose ring of γ-CD. The maximum band at 2930 cm^−1^ comes from the stretching vibrations of the C-H bonds in the CH_2_. A high-intensity doublet, with maximum absorption at 1030 cm^−1^ and 1000 cm^−1^, is assigned to the C-H out-of-plane bending vibrations and the C-OH stretching vibration modes, respectively. The band at 598 cm^−1^ is given by –CH=CH- bending [34,35].

#### 3.3.2. X-ray Diffractometry (XRD)

**Figure 4:**
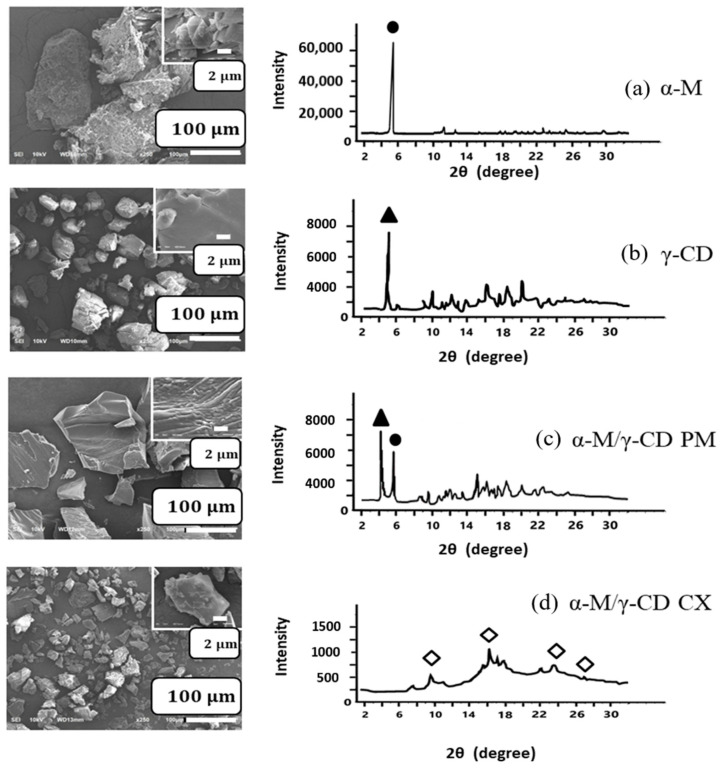
Morphology and XRD pattern.

The crystallinity of intact α-M was 91.1% with the specific peak at 5.890. The crystallinity of intact γ-CD was 62.6% with the specific peak at 5.19. These peaks still remain in α-M/γ-CD PM at 5.14 and 5.95. In contrast, both of the peaks disappeared, and the crystallinity of α-M was decreased into 28.2% in α-M/γ-CD CX; the peak was seen around 16–18 with an intensity of about 1000. This indicates that α-M formed a complex with γ-CD (Figure 4).

The crystallinity of α-M, γ-CD, α-M/γ-CD PM, and α-M/γ-CD CX was examined using PXRD (Figure 4). Intact α-M gives the diffraction peak at 2θ = 5.89°, and intact γ-CD gives the diffraction peak at 2θ = 5.19°. Both of these peaks can be detected at α-M/γ-CD PM at 5.14° and 5.95°. In contrast, the two characterized peaks have disappeared in the α-M/γ-CD CX, resulting in a halo pattern, with the peaks at 10.95°, 16.44°, 22.63°, and 27.33° at low intensity (empty diamond). The solubilization process affected the crystallinity, facilitating the formation of the amorphous structure of the complex, and establishing the new XRD peaks [19,36]. These new peaks are specific to inclusion complexes of γ-CD and a guest molecule [37], suggesting that the inclusion complex was formed in this study.

#### 3.3.3. Scanning Electron Microscopy (SEM)

SEM was performed to analyze the morphology of each sample (Figure 4). The surface of α-M was rough, and the particle size was around 150 μm. The surface of γ-CD was smooth and irregularly shaped, with a particle size less than 100 μm. In the α-M/γ-CD PM (1/1), both particles of α-M and γ-CD can be detected. The cubic grains were seen in the α-M/γ-CD CX, with a particle size around 50 μm and the same smooth surface as for γ-CD.

The surface of α-M was rough, and the particle size was around 150 μm (Figure 4). The surface of γ-CD was smooth and irregularly shaped, with a particle size less than 100 μm. In the α-M/γ-CD PM (1/1), both particles of α-M and γ-CD appeared. As a result of mixing the α-M and γ-CD solutions, a pale yellow powder was obtained, while in the SEM analyses the cubic grains in which fine particles aggregated appeared in the α-M/γ-CD CX. This phenomenon was previously reported about the complex formation of budenoside/γ-CD. The last study findings suggest that cogrinding and coprecipitation resulted in the formation of an inclusion complex and that this influenced the particle diameter and particle surface [34]. Our study found that the crystallinity of α-M/γ-CD CX was 28.2%, while the intact α-M and γ–CD were 91.1% and 62.6%, respectively. The agglomeration of α-M/γ-CD CX indicates the presence of an amorphous state in the complex, responding to the optimum complex formation [38,39].

#### 3.3.4. NMR Spectrometry

The NMR spectra of α-M, γ-CD, α-M/γ-CD PM, and α-M/γ-CD CX were obtained using NMR Spectrometry 500 Hz JEOL, JNM ECA 500. The analysis was performed for proton (H-NMR), C-NMR, and HMBC.

**Figure 5:**
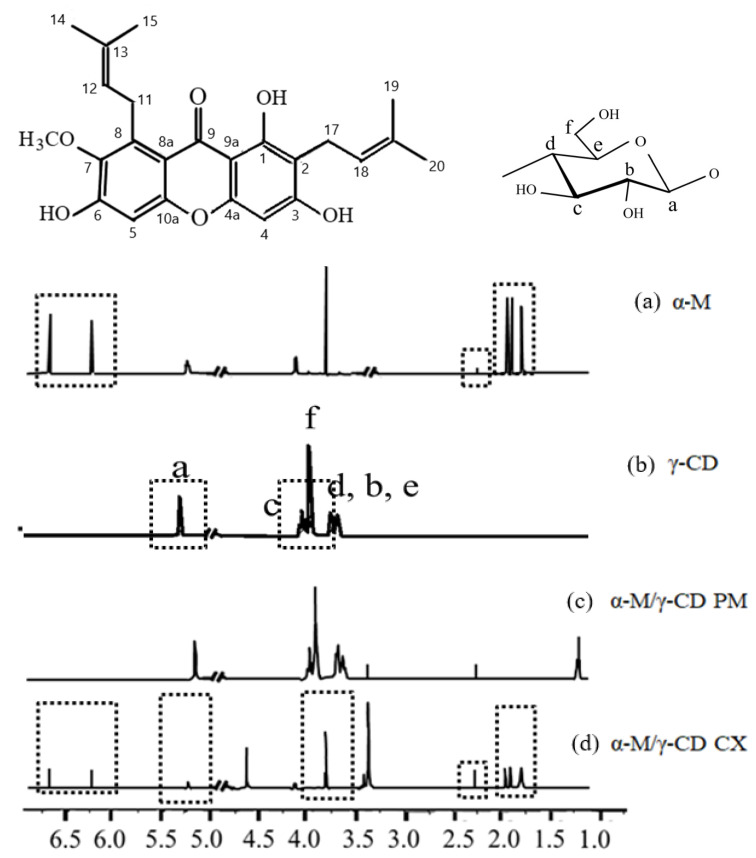
H-NMR spectrum.

1H NMR spectra of α-M, γ-CD, α-M/γ-CD PM, and α-M/γ-CD CX are shown in Figure 5. In α-M, the signal at 6–6.5 ppm is given from an aromatic ring of A and C with hydroxyl groups. A singlet and doublet shifting appeared at 1.671 ppm for the alkyl groups, while the α to carbonyl groups were seen at 2.155 ppm (C is next to C=O). The same peak appears in the α-M/γ-CD CX. These results indicates that the inclusion complex of α-M and γ-CD was formed. Several peaks of α-M and γ-CD disappeared in the α-M/γ-CD PM spectrum [19]. The triplet spectrum indicates that a hydrogen bond was formed near two of the hydrogen atoms. The peak at 3.5–4.0 ppm was given by Cα, which is attached to oxygen. In the other chemical shift, the peak at around 6.5 ppm disappeared, while the other peak was the same as γ-CD [19,25].

The signal of a,b,c,d and e, given by the proton in glucopyranose monomer of γ-CD at a,b,c,d and e position. The integration of the overlapped peaks was used to calculate the molar ratio of α-M and γ-CD in the complex. The result emphasized the Job plot, which showed the molar ratio of this complex is 1:1. The chemical shift of α-M/γ-CD CX was influenced by the guest molecule (α-M) entering into the hydrophobic cavity. This phenomenon indicates that the complex of α-M/γ-CD CX was formed [16,17,18].

**Figure 6:**
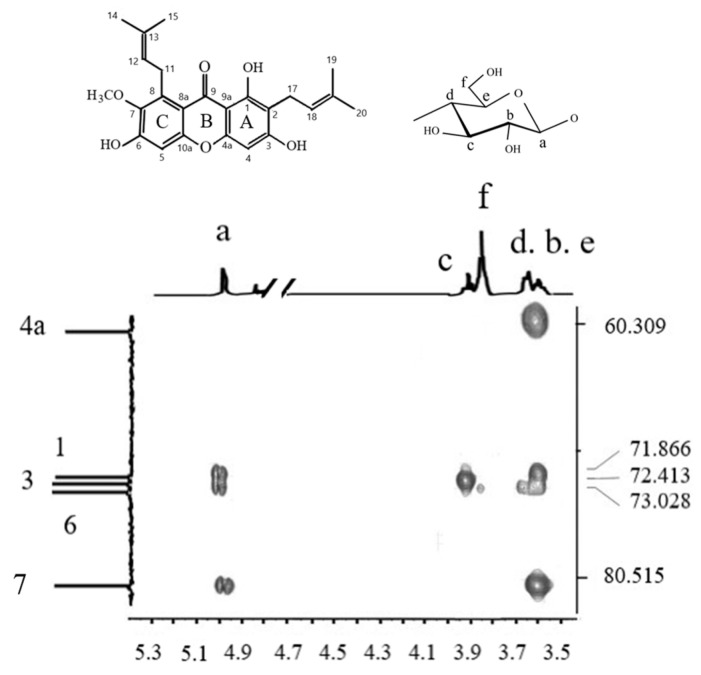
HMBC spectrum of α-M/γ-CD CX.

The study continued by using C-NMR and HMBC to investigate the molecular interaction of α-M and γ-CD. The HMBC spectrum of α-M/γ-CD CX is given in Figure 6.

A hydrogen bond was found at the hydroxyl group of α-M, which interacted with the hydrogen ion of the γ-CD molecule. The signal of a, b, c, d and e, given by the proton in glucopyranose monomer of γ-CD at a, b, c, d and e position. The signal 1, 3, 4a, 6 and 7 given by the carbon of α-M molecule at the same postion. The interaction of α-M and γ-CD shows the interaction between the proton (H) from the hydroxyl groups with the C of the methoxy groups. The hydrogen bond was found at the hydroxy group at the A and C ring of α-M, which interacted with the hydrogen ion at a, b, c, and d of the γ-CD molecule. The interaction occurred at C 4a of α-M with a hydrogen atom at the d position of γ-CD. Two other signals appear at C7 of α-M, with hydrogen at the a and d position of γ-CD. This interaction describes the successful complex formation of α-M and γ-CD [19,25].

### 3.4. Thermodynamic Study of α-M/γ-CD CX Formation

**Figure 7:**
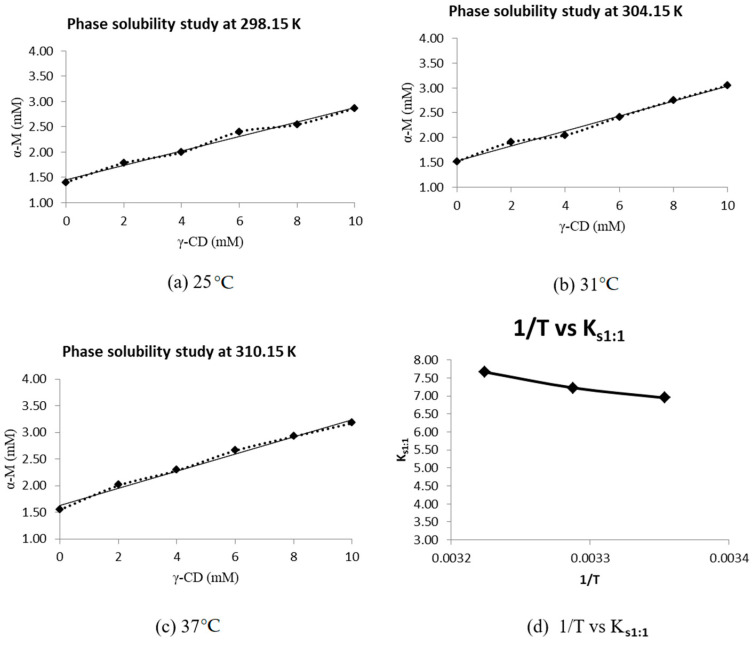
Thermodynamic study of α-Mangostin/γ-Cyclodextrin complex (α-M/γ-CD).

Thermodynamic studies of α-M/γ-CD CX formation were undertaken to study the complex formation process. This procedure was carried out at temperatures of 25 °C, 31 °C, and 37 °C. The stability constant (*K_s_*) in each temperature was calculated by using Equation (1) by using the slope of the graph in each temperature (Figure 7a–c). ∆*G* was calculated by Equation (3) and ∆H is the slope of the diagram in Figure 7d.

The thermodynamic study of α-M/γ-CD CX formation has an energy of complexation of about −5.019 ± 0.224 kcal/mol, and ΔH is −5.520 kcal/mol. An in silico study using the semi-empirical quantum parameterization method 6 (PM6) yielded an energy of complexation of −5.68 kcal/mol, while the parameterization method 7 (PM7) gave an energy of complexation of 5.29 kcal/mmol [1].

The thermodynamic study of α-M/γ-CD CX formation gave an energy of complexation of about −5.019 ± 0.224 kcal/mol and ΔH of −5.520 kcal/mol. The in silico study using PM6 yielded an energy of complexation of −5.68 kcal/mol, while the PM7 method gave an energy of complexation of 5.29 kcal/mmol [1]. 

The in silico study showed that α-M molecules were included in the inclusion complex with γ-CD. In the latest study, PM6 molecular docking indicated that the O4 of γ-CD attached with the hydroxyl groups at C3 and C6 in α-M, while PM7 resulted in the interaction of hydroxyl groups in γ-CD with the methoxy groups at the C ring and the carbonyl group at the B ring of α-M [1].

α-M is able to form an inclusion complex with α-, β-, and γ-CD. The in silico study using PM7 found that the inclusion complex of α-M with γ-CD is the most favorable, because this complex has the lowest negative value of free binding energy (ΔG), −5.68 kcal/mol, while a higher energy was needed for α- and β-cyclodextrin inclusion complexes (−4.76 and −5.02 kcal/mol, respectively) [1]. The calculated experimental free binding energy (ΔG) is −5.019 ± 0.224 kcal/mol.

## 4. Conclusions

The Job plot diagram and H-NMR revealed that α-M/γ-CD CX has formed as an inclusion complex at a stoichiometry ratio of 1:1. Analysis by FTIR, XRD, and NMR (proton, carbon, and HMBC) corroborates that the complex was formed by the molecular interaction of α-M and γ-CD, resulting in an amorphous complex with the solubility improvement and different physical properties compared with α-M. Furthermore, the thermodynamic study exhibited that the Gibbs energy of the complex formation was negative, indicating that this complex was formed spontaneously.

## Data Availability

Not applicable.
